# Effect of photobiomodulation on pain and quality of life in fibromyalgia syndrome: a systematic review

**DOI:** 10.1007/s10103-026-04930-4

**Published:** 2026-06-23

**Authors:** Swathi G A, G. Arun Maiya, Shiran Shetty, Heidi Abrahamse, Rajagopal Kadavigere, Shyamasunder Bhat N, Shivashankara K.N.

**Affiliations:** 1 Department of Physiotherapy, Manipal College of Health Professions, Manipal Academy of Higher Education, Manipal, India; 2 Department of Gastroenterology and Hepatology, Kasturba Medical College, Manipal Academy of Higher Education, Manipal, India; 3 Laser Research Centre, Faculty of Health Sciences, University of Johannesburg, Johannesburg, South Africa; 4 Department of Radiodiagnosis, Kasturba Medical College, Manipal Academy of Higher Education, Manipal, India; 5 Department of Orthopaedics, Kasturba Medical College, Manipal Academy of Higher Education, Manipal, India; 6 Department of Medicine, Kasturba Medical College, Manipal Academy of Higher Education, Manipal, India

**Keywords:** Chronic fatigue syndrome, Photobiomodulation, Laser phototherapy, Low-level light therapy, Chronic pain, Pain, Health-related quality of life, Systematic review, Tender points

## Abstract

Fibromyalgia Syndrome (FMS) is a chronic pain disorder characterized by widespread pain and central sensitization that significantly impacts the quality of life (QoL). For management to be effective, a multidisciplinary approach to care is typically required. Photobiomodulation therapy (PBMT), a non-pharmacological treatment, has garnered attention lately, though its clinical relevance and applications are not well defined. The objective of this review was to assess the effectiveness of PBMT in reducing FMS symptoms. This systematic review was registered at PROSPERO (CRD420251084730) and conducted following PRISMA (Preferred Reporting Items for Systematic Reviews and Meta-Analyses) reporting guidelines. A total of seven randomized controlled trials were identified following an extensive literature search across various databases, including PubMed, Scopus, Web of Science, the Cochrane Library, Embase, Ovid and ProQuest. To evaluate the methodological quality of these studies, the Cochrane Risk of Bias (RoB 2.0) tool was applied. PBMT demonstrated consistent short-term reductions in pain intensity and improvements in QoL. Additional positive effects on sleep quality and psychological well-being were observed, indicating that PBMT may provide additional therapeutic benefits beyond pain reduction, including improvements in sleep quality and psychological well-being. PBMT has shown promise as a safe, non-pharmacological adjunct therapy that may provide short-term improvements in pain levels and QoL, but substantial heterogeneity limits generalizability. Clinical trials with large samples and standardized methodologies should be conducted to better clarify the role of PBMT in multidisciplinary therapy for FMS.

## Introduction

Fibromyalgia Syndrome (FMS) is a chronic condition that leads to widespread pain and tenderness throughout the body. This happens because the nervous system becomes overly sensitive and reacts strongly to pain signals [1, 2]. In addition to chronic pain, fibromyalgia is a complex disorder frequently accompanied by fatigue, morning stiffness, disrupted sleep, and cognitive problems, such as memory lapses and poor concentration [3, 4].

This condition is often diagnosed in women aged 20–50 years, although men, adolescents, and older adults can also be affected [5–7]. It is believed to have its roots in a confluence of mood-related disorders, psychological stress, and hereditary susceptibility. In addition to frequently coexisting with rheumatic conditions like systemic lupus erythematosus and rheumatoid arthritis, fibromyalgia syndrome has occasionally been connected to infectious agents like the Epstein-Barr virus [8–10]. Its prevalence is believed to be between 2.6% and 6.6% worldwide, with greater rates among women. Nonetheless, reports of the frequency in India range from 0.05 to 2%, which is relatively low [11, 12].

Patients’ QoL is significantly impacted by fibromyalgia, which affects their social, emotional, and physical functioning. Due to frequent medical visits and decreased productivity at work, it also presents a substantial financial burden [13, 14]. A multidisciplinary approach to treatment typically includes cognitive-behavioural therapy, exercise regimens, analgesics, antidepressants, anticonvulsants, and patient education [15–17]. These methods can ease symptoms and improve function; however, outcomes often vary, and adherence tends to be poor. Consequently, many patients remain undertreated, generating interest in complementary and non-drug therapies. A placebo response has also been shown to play a substantial role in fibromyalgia interventions, emphasizing the necessity for well-validated and sustainable treatment options for fibromyalgia [18].

Recent research has indicated Photobiomodulation therapy (PBMT) can be utilized as an adjuvant in treating chronic pain syndromes, including fibromyalgia [19, 20]. low-level lasers or light-emitting diodes (LEDs). PBMT produces red to near-infrared light (600–1100 nm). PBMT mainly functions by the absorption of photons by cytochrome c oxidase (CCO) in the mitochondrial respiratory chain. By altering cellular activity, this mechanism facilitates tissue healing, reduces pain, and inflammation [21]. Research indicates that photobiomodulation enhances mitochondrial ATP production, decreases oxidative stress, and influences the expression of genes involved in pain pathways [22]. As fibromyalgia is associated with mitochondrial dysfunction, neuroinflammation, central sensitization, and autonomic imbalance, these mechanisms provide a physiologically coherent rationale for the observed improvements in pain, fatigue, and related psychological manifestations. Rather than directly targeting psychological constructs, PBMT may reduce psychological distress indirectly through modulation of pain intensity, autonomic balance, and neuroinflammatory signalling, which are known contributors to fear avoidance behaviour and reduced self-efficacy [21].

The multifaceted nature of fibromyalgia, marked by diverse symptom patterns and underlying mechanisms, has led to inconclusive findings regarding the efficacy of photobiomodulation. This challenge is compounded by the considerable variability in the reported photobiomodulation protocols, with uncertainties surrounding the ideal wavelengths, session length, treatment frequency, and long-term safety [22]. Despite promising findings, the clinical effectiveness of PBMT in fibromyalgia remains uncertain because of variability in treatment protocols, including wavelength, dosage, treatment frequency, and duration. Inconsistencies in outcome measures and methodological quality across studies further limit comparability and clinical interpretation. Therefore, a systematic review is needed to critically evaluate the available evidence and clarify the role of PBMT in fibromyalgia management.

Therefore, this systematic review aimed to assess the effectiveness of photobiomodulation in FMS, especially in reducing pain and improving QoL, as well as exploring possible influencing factors.

## Materials and methods

### Data source and search strategy

The protocol was prospectively registered on PROSPERO (CRD420251084730), and the review was conducted in accordance with the PRISMA guidelines for systematic reviews [23]. A thorough literature search was conducted from January 1, 2025, to July 31, 2025, to identify relevant studies evaluating the effects of photobiomodulation on pain and QoL in patients with fibromyalgia. The databases searched were PubMed, Scopus, Web of Science, Cochrane, Embase, Ovid MEDLINE, and ProQuest for grey literature. No other filters were applied.

The following search equation was used in PubMed:

fibromyalgia[MeSH Terms] OR “fibromyalgia syndrome” OR “muscular rheumatism” OR “primary fibromyalgia” OR “secondary fibromyalgia” OR “fibrositis” AND “low-level light therapy“[MeSH Terms] OR “Photobiomodulation” OR “PBMT” OR “laser phototherapy” OR “low-level laser therapy” OR “laser biostimulation” AND “Pain“[Mesh] OR “Pain Management“[Mesh] OR “Musculoskeletal Pain“[Mesh] OR “Chronic Pain“[Mesh] AND “quality of life“[MeSH Terms]) OR “health-related quality of life” OR “HRQoL”.

Equivalent strategies were applied to the other databases. The searches were independently conducted by two reviewers (SGA and AGM). All data extracted from the databases were imported into the Rayyan software [24]. The SGA screened the original titles and abstracts, eliminating duplicate studies. Both reviewers independently evaluated the full texts and extracted pertinent information from eligible articles; AGM resolved any disagreements regarding study selection.

### Selection of studies

Studies that satisfied the following requirements were considered eligible: (1) Research designs, including only randomized controlled trials (RCTs) to ensure methodological rigor; (2) availability of full text; (3) publication in the English language; (4) participants were diagnosed with fibromyalgia syndrome; (5) photobiomodulation was used as the primary intervention, and (6) outcomes were reported on at least one primary measure: pain intensity or QoL.

The exclusion criteria included: (1) case reports/series, editorials, commentaries, letters, or systematic/narrative reviews; (2) studies that were not published in English or that did not have full texts available; (3) studies that did not use PBMT as the primary treatment; and (4) studies that did not measure QoL, pain intensity, or pain pressure threshold (PPT).

### Data extraction

SGA and AGM separately extracted data using a standardized table organized according to the Population, Intervention, Comparison, Outcome (PICO) framework. PBMT parameters (e.g., wavelength, dose, treatment duration, frequency, application site, and number of sessions) were reported according to Hamblin’s article [25]. Intervention and control groups, and study characteristics (author, year, country, study design, objectives, key outcomes assessed, sample size, and sex distribution) were among the data gathered. The extraction table was piloted prior to its use in all studies, while the data extraction template was designed based on the guidelines outlined in the Cochrane Handbook for Systematic Reviews of Interventions (v. 5.1.0).

### Methodological quality assessment

#### Risk of bias assessment: ROB 2.0

The Risk of Bias tool, used for analysing randomized controlled trials, was used by two reviewers (SGA and AGM) to evaluate the included randomized clinical trials [26]. The RoB tool considers the following five areas/domains: randomization process, deviations from intended interventions, missing outcome data, outcome measurement, and selection of reported results. Differences were discussed between SGA and AGM, and in case of disagreement, resolved by AGM.

#### Quality of evidence: GRADE

The assessment of the quality of the evidence was based on five domains, which included the study design, imprecision, indirectness, inconsistency, and publication bias, according to the Grading of Recommendations, Assessment, Development, and Evaluation (GRADE) framework [27]. It resulted in the categorization of the evidence into high, moderate, low, or very low levels of certainty.

## Results

### Study selection

In total, 1,448 records were retrieved from various databases such as PubMed (63), Scopus (134), Cochrane (49), Embase (125), Web of Science (37), Ovid (55), and ProQuest (985). Out of these records, 1224 duplicates were removed, leaving a total of 224 records to be screened through title and abstract screening. Of these, 202 were excluded based on predefined eligibility criteria, resulting in twenty-two articles being selected for full-text evaluation.

**Fig. 1:**
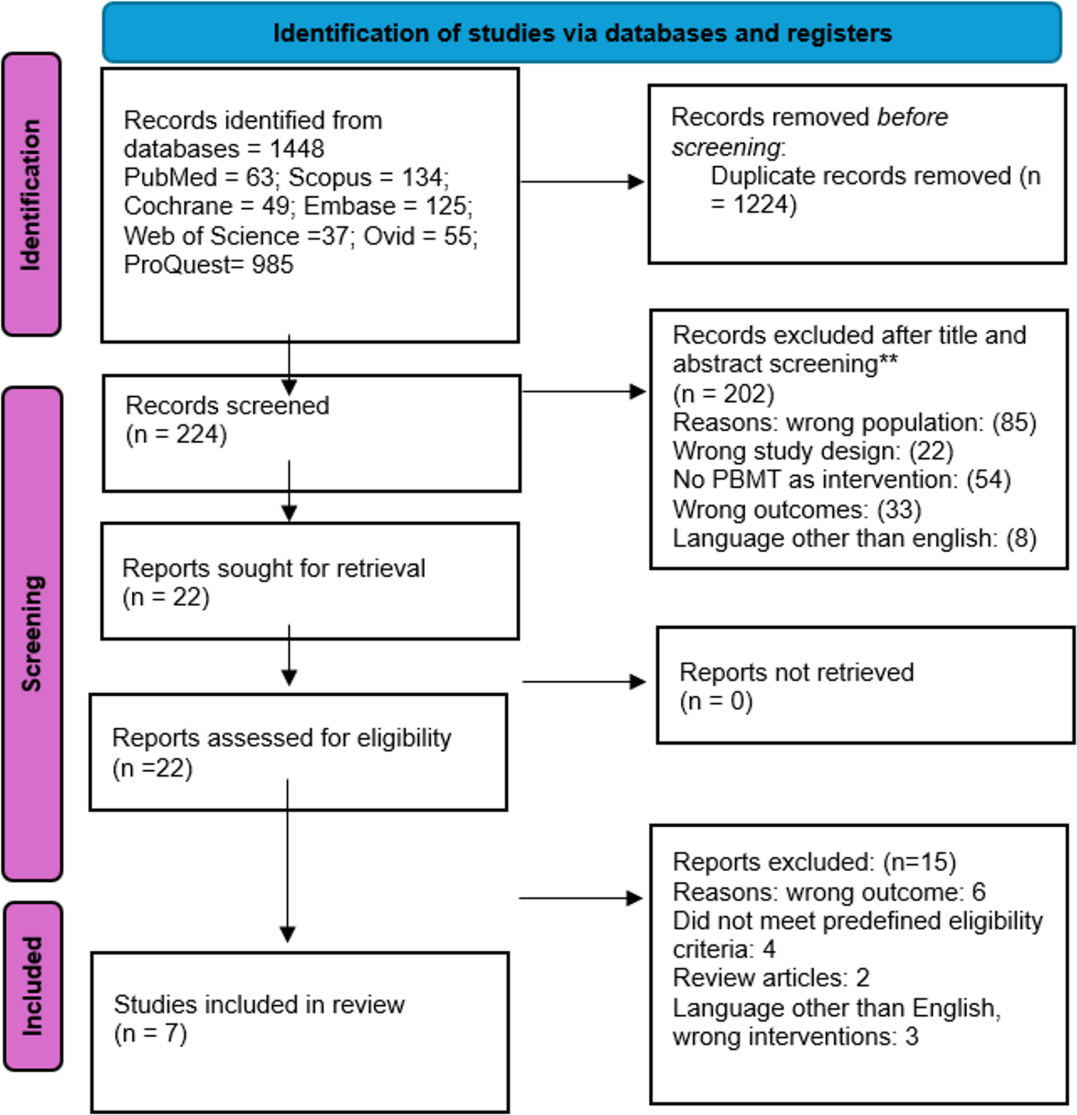
PRISMA 2020 flow diagram represents studies included in this systematic review. PRISMA 2020 flow diagram outlining the process of study identification, screening, eligibility assessment, and final inclusion evaluating photobiomodulation in fibromyalgia syndrome. Records were excluded based on predefined eligibility criteria (population, intervention, outcomes, and study design)

Out of the total number of 22 full-text articles that were examined, 15 were excluded. Ultimately, seven randomized controlled trials met all eligibility requirements and were added to the evaluation. The process of article selection is briefly described in the PRISMA 2020 flow diagram shown in Fig. 1.

### Characteristics of the included studies

A total of seven randomized controlled trials involved 495 participants, who were mostly female subjects aged between 30 and 55, and had been clinically diagnosed with fibromyalgia using the American College of Rheumatology criteria. Details about the selected studies are given in Table 1 below.

**Table 1:** Characteristics of the included studies

Author, Year	Country	Study design	Participants	Intervention	Comparison
Vassao et al. (2024) [28]	Brazil	Double-blinded randomized controlled trial	*N* = 51 women with fibromyalgia	PBMT + Aerobic Exercise	PBMT only; Exercise only; Control
Maciel et al. (2018) [29]	Brazil	Double-blind randomized clinical trial	*N* = 22 women with fibromyalgia	Low-Level Laser Therapy	Placebo laser + exercise
Silva et al. (2018) [30]	Brazil	Randomized, blinded controlled trial	*N* = 160 individuals with fibromyalgia	Cluster PBMT	Exercise alone
Ribeiro et al. (2023) [31]	Brazil	Triple-blinded, randomized, placebo-controlled trial	*N* = 90 women with fibromyalgia	PBMT combined with a static magnetic field	Placebo PBMT-sMF (device off, red LED 1 mW visual only)
Panton et al. (2013) [32]	USA	Randomized, sham-controlled trial	*N* = 38 women with fibromyalgia	Class IV High-Power Laser	Sham laser
Gur et al. (2002) [33]	Turkey	Single-blind placebo-controlled trial	*N* = 40 individuals with fibromyalgia	Ga-As Laser	Placebo laser
Ruaro et al. (2014) [34]	Brazil	Randomized placebo-controlled trial	*N* = 20 women with fibromyalgia	GaAlAs Laser (670 nm)	Placebo laser

Entire characteristics of included studies in this systematic review, including study design, sample size, intervention type, control condition, duration, and primary outcomes assessed. PBMT-sMF-Photobiomodulation therapy with static magnetic field, GaAlAs- Gallium, Aluminum, Arsenic, cluster PBMT- cluster photobiomodulation therapy, Ga-As Laser- Gallium, Arsenic laser

Three studies were double blind randomized controlled trials (RCTs) [28–30]. The remaining four were randomized placebo-controlled trials [31–34]. Geographic distribution includes Brazil (5 studies), the USA (1 study), and Turkey (1 study). Six studies evaluated low-level PBMT (CLASS III devices or LED systems, < 500mW). One study [32] evaluated a high-power class IV laser protocol. Due to substantial differences in power output (> 500 mW). Comparative interventions, such as standard medical care [28, 30–33], exercise [29] and sham/placebo photobiomodulation [34].

### PBMT protocol characteristics

PBMT protocols varied across the included studies with respect to device type, wavelength, energy dose, treatment duration, and application sites. Devices included low-level laser therapy (LLLT), LED systems, cluster probes, and one Class IV high-power laser system. Reported wavelengths ranged from 630 to 905 nm, with radiant exposures between 2 and 142.8 J/cm². Treatment frequency ranged from 2 to 3 sessions per week over periods of 2–12 weeks, and the number of treated tender points or muscle sites ranged from 7 to 18. Detailed PBMT parameters are summarized in Table 2.

**Table 2:** Photobiomodulation parameters used across the included trials

Study	Device	Wavelength (nm)	Output power (mW)	Exposure time	Radiant exposure (J/cm²)	Dose (J)	No.of points	Area irradiated cm^2^	Application technique	Sessions
Vassao et al. (2024). [28]	Antares^®^ cluster probe with seven diodes	808	100	50/80/100 s	Not reported by authors	20/32/40 J (total)	10	0.07	Contact	3 sessions/week for 12 weeks
Maciel et al. (2018). [29]	DMC Photon Laser III	808	100	40s per site Total 680s	142.85 J/cm²	4 J per Site	17	0.028	Contact	24 (8 weeks)
Silva et al. (2018). [30]	Pain Away/Pain Cure™ nine-diode cluster device	905, 640, 875	0.9, 15, 17.5	300s per point	0.075 5 per site 5.83 per site	Total 39.3 J per site	10	0.4, 0.9/site 0.9/site Total = 4	Contact	20 (10 weeks)
Ribeiro et al. (2023). [31]	Fibrolux^tm^ therapy system	905 (laser) 850 (IR LED) 630 (red LED)	5, 37.50, 25	120s per region	1.87, 8.04, 3.53	0.60,4.50,3 Total (60 J per region)	18	0.32, 0.56, 0.85	Contact	3 sessions/week for 3 weeks
Panton et al. (2013). [32]	Class IV Laser LCT 1000	810–980	> 500	60 s per point Total 7 min/session	10.63	600 J per point Total 4200 J per session	8	56.45	Not reported by the authors	8 (4 weeks)
Gur et al. (2002). [33]	Ga-As Laser Class III b	904	11.2	3 min/point	2	Not reported by the authors	18	1	Not reported by the authors	10 (2 weeks)
Ruaro et al. (2014) [34]	GaAlAs Diode Laser	670	20	7s/point Total 504 s	4 J/cm²	Not reported by the authors	18	0.035	Non-contact with a distance of 1 cm	12

Parameters include wavelength, power output, exposure time, radiant exposure, dose, number of points treated, area irradiated, application technique, and total sessions. *PBMT * Photobiomodulation therapy, *nm* nanometers (wavelength), *mW *milliwatts (output power); note that Panton et al. [32] reports > 0.5 W (i.e., > 500 mW), consistent with Class IV classification, *s *seconds, *J/cm² *joules per square centimeter (radiant exposure/energy density), *J* joules (total energy delivered per point, site, or region), *Contact *probe applied directly to the skin, *Non-contact *probe held at a specified distance from the skin Values separated by commas (e.g., 905, 850, 630 nm) indicate multiple wavelengths used simultaneously or in combination within the device (multi-diode/cluster probes)

One study evaluated a Class IV high-power laser protocol [32], which differed from the remaining low-level PBMT studies in terms of output power (> 500 mW), irradiance, and energy delivery. To improve interpretability, findings from this study were analyzed and presented separately throughout the review.

### Summary of outcome measures

All seven trials assessed pain intensity using a VAS or an equivalent scale. Six low-level PBMT studies demonstrated significant reductions in pain compared with baseline or control groups, whereas the single Class IV laser study reported limited pain improvement. Five studies evaluated QoL using FIQ/FIQR or SF-36, with four reporting clinically meaningful improvements. Secondary outcomes included tender point count, pressure pain threshold, fatigue, sleep quality, and functional performance (6-minute walk test and Timed Up and Go). Changes in important outcomes are shown in Table 3 below.

**Table 3:** Key outcome changes following low-level pbmt in included studies

Study	Pain intensity (VAS or equivalent)	QoL (FIQ / SF-36)	Tender points count	Pressure Pain Threshold (PPT)	Other notable outcomes	Notes / combination effect	Adverse events/ safety	Conclusion
Vassao et al. (2024) [28]	↓ 6.8 → 4.2 (~38%) (*p*<0.05)	SF-36: ↑ 38.4 → 55.7 (~45%) (*p*<0.05)	Not reported	Not reported	Improved 6MWT, mobility, and strength	Best results with PBMT + aerobic exercise	No adverse events reported	PBMT combined with exercise enhances treatment outcomes compared with isolated interventions.
Maciel et al. (2018) [29]	Significant VAS reduction	FIQ improvement	Significant reduction	Not reported	Improved strength, flexibility, balance	PBMT + functional exercise; some synergy	No adverse events reported	Combining LLLT and exercise improves pain and functional outcomes; there is no superiority over exercise alone in some measures.
Silva et al. (2018) [30]	Significant VAS reduction	FIQ improvement	Significant reduction	Increased	Reduced fatigue (FSS), stiffness	PBMT + exercise is superior to exercise alone	No adverse events reported	PBMT + exercise improves pain and QoL more than exercise alone.
Ribeiro et al. (2023) [31]	↓ 80.64 → 37.80 (~53%) (*p*<0.0001)	FIQ: ↓ 79.68 → 43.89 (~45%) (*p*<0.001)	↓ 15.29 → 7.29 (~52%) (*p*<0.0001)	Not reported	Reduced tender points, FIQ domains improved	Strongest effect with a static magnetic field	Mild transient pain/tension in 7.8% participants; resolved spontaneously	PBMT-sMF significantly reduces pain, tender points, and fibromyalgia impact compared with placebo.
Gur et al. (2002) [33]	Significant VAS reduction (3.09 → 1.27)	Not the primary outcome	Significant reduction	Increased	Improved sleep, stiffness	Low-power Ga-As laser; short-term benefits	No adverse events reported	Low-power laser therapy reduces pain and improves symptoms in the short term.
Ruaro et al. (2014) [34]	↓ 8.1 → 5.4 (~33%) (*p*<0.001)	FIQ improvement	Not reported	Increased	Reduced McGill Pain scores	GaAlAs laser; significant pain and impact reduction	No adverse events reported	LLLT significantly reduces pain and improves the impact.

Summary of main outcome changes in the seven included randomized controlled trials evaluating PBMT for fibromyalgia syndrome. Arrows indicate direction of change (↓ = reduction/improvement for negative scales; ↑ = improvement for positive scales such as SF-36). Percentages are approximate where exact values were derivable from reported means. *VAS* Visual Analog Scale, *FIQ* Fibromyalgia Impact Questionnaire, *SF-36* Short Form-36, *6MWT* 6-Minute Walk Test, *FSS* Fatigue Severity Scale. “Not reported” indicates the outcome was not a primary or secondary measure in that study

Because of the significant differences in dosimetry and mechanism between Class III and Class IV laser systems, the findings of the Class IV laser study were interpreted separately and not directly compared with low-level PBMT trials. Key outcome changes are summarized in Table 4 for improved comparability across studies.

**Table 4:** Key outcome changes following Class IV laser study

Study	Pain intensity (VAS or equivalent)	QoL (FIQ / SF-36)	Tender points count	Pressure Pain Threshold (PPT)	Other notable outcomes CS-PFP-	Notes / combination effect	Adverse events/ safety	Conclusions
Panton et al. (2013) [32]	Minimal change: 6.1 → 6.2 (~2%)	FIQ: ↓ 62 → 55 (~11%) (p<0.05, modest)	Not reported	Not reported	Improved functional performance	Class IV laser; limited pain relief	No adverse events reported	Class IV laser improved function but did not significantly reduce pain intensity.

The Class IV laser study [32] was analyzed separately due to substantially different irradiance and total energy delivery compared with low-level PBMT*.* *VAS *Visual Analog Scale, *FIQ* Fibromyalgia Impact Questionnaire; SF-36 = Short Form-36, *CS-PFP *Continuous Scale Physical Functional Performance

### Risk of bias assessment (RoB 2)

Risk of Bias in the selected articles was evaluated using the Cochrane Risk of Bias 2 tool, with the findings ranging from low to severe [28–34]. Each study has a different risk of bias. Two studies [28, 34] were rated as having a low risk of bias, whiles two of them raised some concerns [30, 33], and the remaining three were assessed as having a high risk [29, 31, 32].

Domain 1 (randomization process): Five studies were rated as having a low risk; however, Maciel et al. [29], da Silva et al. [30] and Panton et al. [32] raised some concerns due to a limited report on allocation concealment.

Domain 2 (deviations from intended interventions): Only Ruaro et al. [34] scored this domain as low risk. The remaining six studies were at high risk due to insufficient blinding or non-adherence to the protocol.

Domain 3 (missing outcome data): Five studies were rated as low risk, whereas Ribero et al. [31] and Maciel et al. [29] raised concerns due to incomplete follow-up.

Domain 4 (measurement of outcomes): Ruaro et al. [34] and da Silva et al. [30] were rated as low risk. The remaining studies presented some concerns or were classified as high risk because of issues with subjective outcomes and a lack of blinding.

Domain 5 (selection of reported results): Four studies were assessed as low risk, whereas Ribero et al. [31], Maciel et al. [29], and Panton et al. [32] were considered high risk.

**Fig. 2:**
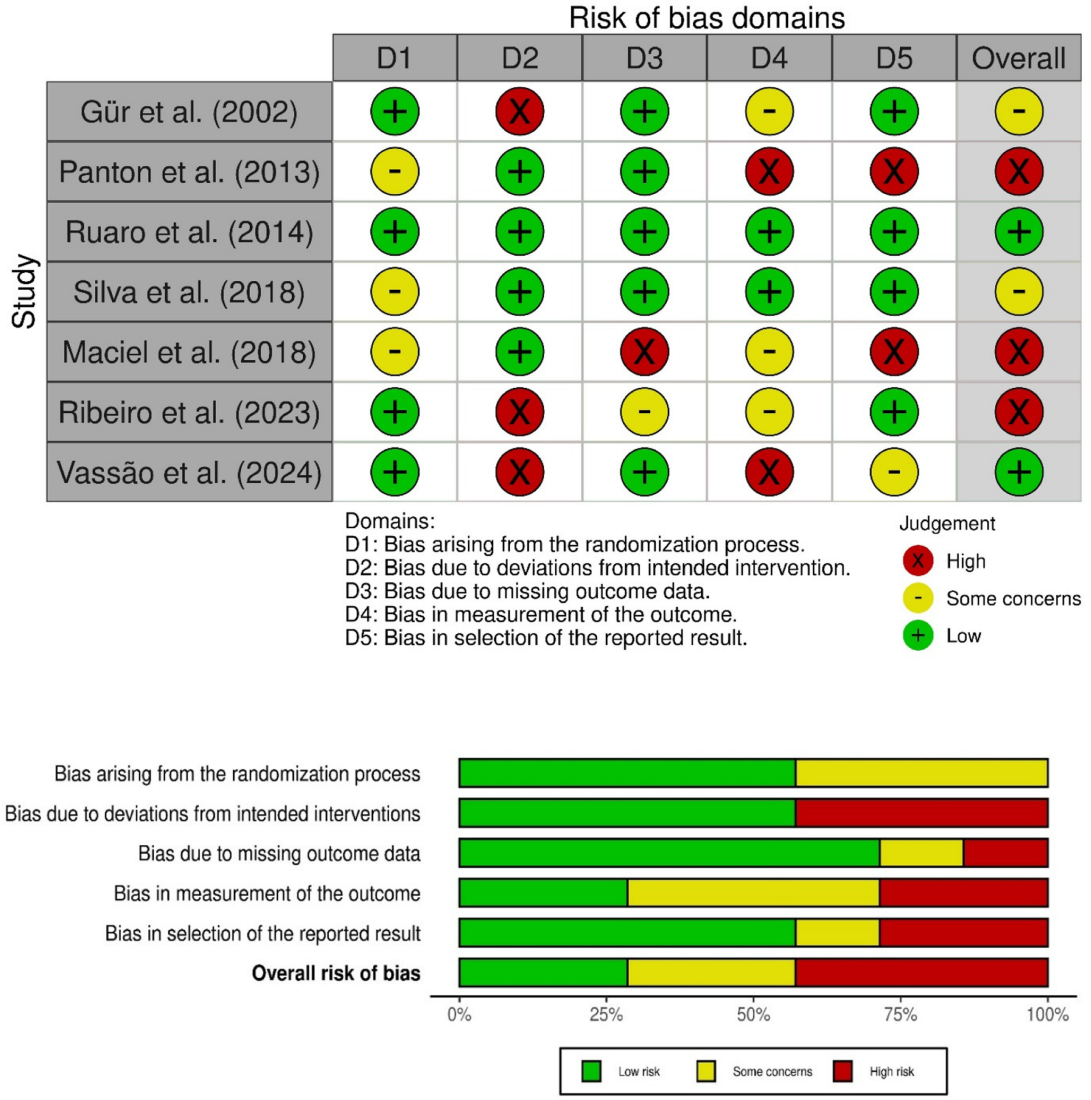
Risk of bias. Risk of bias assessment for the included studies using the Cochrane RoB 2.0 tool. Green, yellow, and red indicate low, some concerns, and high risk, respectively. Domains assessed include randomization process, deviations from interventions, missing outcome data, outcome measurement, and selection of reported results. *A traffic light plot was created via the ‘robvis’ package in R*

Overall, methodological rigor was mixed, with some studies well-designed and others compromised by bias in reporting and blinding. Figure 2 provides a visual summary of the study.

### GRADE recommendation

The GRADE evaluation indicated that most of the outcomes had moderate-to-high levels of certainty of evidence. Pain intensity was significantly reduced in seven studies (*n* = 421) [28–34], leading to moderate certainty evidence in favour of intervention. Five studies (*n* = 359) that evaluated QoL found moderate to great improvements in everyday functioning and general well-being [28–31, 34], suggesting PBMT probably provides short-term clinical benefit in patients with fibromyalgia syndrome. However, certainty was downgraded because of methodological limitations and heterogeneity in PBMT parameters across studies.

A low certainty of evidence resulted from the evaluation of tender point sensitivity in four studies (*n* = 188) [29–31, 33] Downgraded for methodological limitations and imprecision due to small sample sizes. Three studies (*n* = 220) that looked at the pressure pain threshold [30, 33, 34] found that it had improved somewhat, leading to a low certainty of evidence. Downgraded due to methodological limitations and imprecision across small trials.

With few discrepancies and no notable problems with methodological limitations, indirectness, or imprecision, the included studies generally showed a low-to-moderate risk of bias. With low to moderate certainty of evidence across multiple outcomes, these findings lend credence to the idea that photobiomodulation is a feasible non-pharmacological treatment for fibromyalgia syndrome. Table 5 provides a thorough synopsis of the GRADE evaluation.

**Table 5:** GRADE assessment of the certainty of evidence across primary outcomes

Outcomes	No. of studies	Risk of bias	Inconsistency	Indirectness	Imprecision	Publication bias	Certainty of evidence
Pain (VAS)	7	Serious	Not serious	Not serious	Not serious	None	⨁⨁⨁◯ Moderate
Tender Point Count	5	Serious	Not serious	Not serious	Serious	None	⨁⨁◯◯ Low
Pressure Pain Threshold	3	Serious	Not serious	Not serious	Serious	None	⨁⨁◯◯ Low
QoL (FIQ/SF-36)	5	Serious	Not serious	Not serious	Not serious	None	⨁⨁⨁◯ Moderate

This table evaluates risk of bias, inconsistency, indirectness, imprecision, and potential publication bias for outcomes like Pain (VAS), tender point, pressure pain threshold, and QoL (FIQ/SF-36). Certainty levels: high, moderate, low, very low. Additional elements considered are the number of studies or subjects, methodological quality, and the overall strength of the evidence

### Data synthesis

Due to significant variability in wavelength, irradiance, radiant exposure (J/cm²), and treatment frequency, quantitative pooling was not conducted.

Pain: Six studies reported consistent short-term pain reductions of 30–55%. Gur et al. [33] noted a decrease in Visual Analog Scale (VAS) scores from 3.09 to 1.27 (*p* < 0.05) with Ga-As lasers. Ruaro et al. [34] found a decrease from 8.1 to 5.4 (*p* < 0.001) with GaAlAs lasers. Ribeiro et al. [31] showed a significant reduction with photobiomodulation-sMF, VAS scores decreasing from 80.64 to 37.80 (*p* < 0.0001). Studies combining exercise with photobiomodulation [28–30] demonstrated enhanced benefits, including muscle strength and QoL improvements. In contrast, Panton et al. [32] reported minimal change in VAS scores with Class IV laser therapy.

QoL: Improvements in quality of life (QoL) correlated with pain reductions, particularly in physical function and vitality. Maciel et al. [29] and Silva et al. [30] reported functional gains contributing to QoL, though FIQ or SF-36 scores were not always primary outcomes. Ribeiro et al. [31] indicated significant reductions in both FIQ and VAS scores. Notably, Vassao et al. [28] showed SF-36 scores improved from 38.4 to 55.7 (*p* < 0.05). Panton et al. [32] reported a moderate yet non-significant improvement in FIQ.

**Fig. 3:**
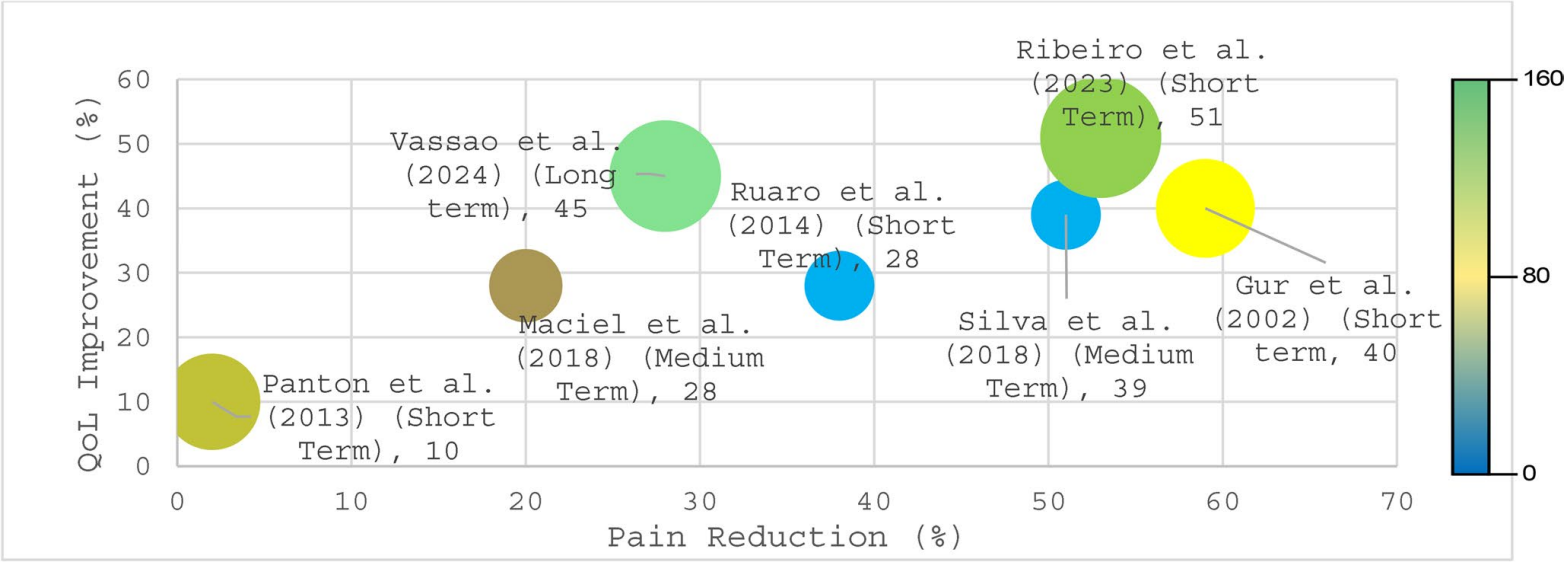
Bubble chart summarizing the percentage change in pain and QoL following PBMT in fibromyalgia syndrome. The x-axis represents the percentage reduction in pain (based on VAS or equivalent scales), while the y-axis reflects the improvement in QoL (based on FIQ/SF-36 scores). Bubble size indicates study sample size; colors differentiate individuals’ studies. Data derived from the seven included trials. *This bubble chart was created via Microsoft Excel*

Mechanosensitivity: Increased pressure pain thresholds (PPT) and reduced tender points suggest modulation of nociceptors and central sensitization. Gur et al. [33] and Silva et al. [30] found increased PPT, while Ribeiro et al. [31] reported a decrease in painful points from 15.29 to 7.29 (*p* < 0.0001). Maciel et al. [29] reported tender point relief and strength gains. Overall, findings indicate short-term symptomatic improvement, but variability in dosimetry challenges definitive parameter recommendations. Further details are available in Fig. 3.

### Safety and adverse effects

Safety outcomes were inconsistently reported across studies. No serious adverse events were identified in any included trial. Ribeiro et al. [31] was the only study that systematically reported adverse effects, documenting mild transient increases in pain or muscular tension in 7.8% of participants receiving active PBMT-sMF. These symptoms are resolved spontaneously without treatment discontinuation. The remaining studies did not report adverse events, limiting conclusions regarding long-term safety. Overall, available evidence suggests that PBMT is generally well tolerated in the short term.

## Discussion

This systematic review analyses existing evidence derived from seven randomized controlled trials that examine the effect of PBMT on pain and QoL in people with fibromyalgia syndrome. Overall, there were consistent short-term reductions in pain intensity and improvements in health-related QoL, particularly in physical function, vitality, and mental health domains, compared to placebo, sham interventions, or standard care. Moderate to high certainty evidence supported analgesic effects, while improvements in functional capacity and multidimensional symptom burden were also observed. However, due to substantial heterogeneity in PBMT parameters and outcomes reporting, a meta-analysis was not conducted.

### Interpretation of main findings

Pain reduction was the most consistent finding across the included studies, with six of seven trials reporting significant improvements in pain intensity compared to baseline or control conditions [28–34]. Increases in pressure pain threshold and reduction in tender point count further support the potential analgesic effects of PBMT in FMS [1, 2]. These findings are clinically relevant because central sensitization and amplified nociceptive processing are key features of fibromyalgia pathophysiology.

The observed clinical benefits are biologically plausible. PBMT is believed to act through photon absorption by cytochrome c oxidase within the mitochondrial respiratory chain, resulting in increased ATP production and modulation of oxidative stress [33]. PBMT also demonstrates anti-inflammatory effects by regulating the levels of pro-inflammatory cytokines, such as TNF-α and IL-1β, as well as anti-inflammatory cytokines. Additionally, it modulates neurotransmitter release and stimulates the release of endogenous opioids, which can mediate analgesia [35]. Since mitochondrial dysfunction, neuroinflammation, and autonomic dysfunction have been proposed to contribute to fibromyalgia pathophysiology [36]. These mechanisms offer a plausible explanation for the observed clinical response.

Notably, the single study employing a Class IV high-power laser yielded only modest functional gains without significant pain reduction, contrasting with the more consistent analgesic effects observed in low-level PBMT using a total power of ≤ 500 mW [32]. Because Class IV laser systems operate at substantially higher power outputs and irradiance levels than low-level PBMT devices, their physiological effects may differ and could include photothermal mechanisms. For this reason, findings from the Class IV laser study were interpreted separately in the present review.

While photobiomodulation is typically applied peripherally to tender points or muscular regions, its effects may extend beyond local tissues. FMS is primarily characterized by central sensitization, involving amplification of nociceptive signalling within the central nervous system [36]. This raises the important question of how peripherally applied PBMT can influence centrally mediated pain. Emerging evidence suggests several bridging pathways. Peripheral nociceptor activity is known to contribute to the maintenance of central sensitization through continuous afferent input. First, PBMT reduces peripheral nociceptor hyperexcitability and local inflammation (downregulation of TNF-α and IL-1β), thereby decreasing afferent barrage to the dorsal horn and indirectly attenuating central windup. Second, PBMT modulates mitochondrial function and oxidative stress, which indirectly reduces nociceptive input to the spinal cord and higher pain centres. Third, improvements in autonomic balance and sleep quality following PBMT may further dampen central amplification of pain signals [35] Nevertheless, direct evidence of PBMT altering brain-level central sensitization markers (e.g., via functional neuroimaging) in fibromyalgia patients remains scarce. This peripheral modulation may therefore contribute to downregulation of central sensitization mechanisms, potentially explaining the observed reductions in pain intensity and mechanosensitivity reported in severe trials [17].

### QoL and functional outcomes

Improvements in QoL were reported in five studies using validated instruments such as FIQ/FIQR and SF-36 [28–34]. The greatest benefit was noted in the areas of physical function, vitality, and mental health domains. These findings are consistent with the idea that pain reduction can indirectly improve mood, fatigue, and functional status [37, 38].

The addition of exercises to PBMT seemed to provide additive effects in some studies [28–34]. According to EULAR recommendations 2017, exercise is strongly recommended in fibromyalgia management guidelines [13, 14], and PBMT may help to improve exercise tolerance by decreasing the pain and stiffness, thereby facilitating adherence to an exercise program. However, because of the heterogeneity of cointerventions, the independent contribution of PBMT cannot be fully isolated [39, 40].Emerging evidence suggests that PBMT may positively influence mood by directly addressing chronic pain and fatigue, showing its potential as a versatile therapeutic tool [40].

### Methodological considerations and heterogeneity

Despite the promising results, there was considerable heterogeneity in the studies regarding the parameters of PBMT, including wavelength, radiant exposure, number of treated sites, session frequency, and treatment duration. There was also some variability in outcome measures, time points of assessment, and the way results were reported. This made it difficult to compare the studies directly and prevented the performance of a meta-analysis, which requires quantitative data pooling.

Risk of bias assessments using RoB 2.0 showed variable methodological quality, with some studies being at low risk of bias and others having concerns about bias due to blinding, allocation concealment, and selective reporting [26]. The placebo effects have been widely observed in fibromyalgia trials [18], emphasizing the importance of rigorous blinding in future PBMT research.

Although the GRADE evaluation showed moderate-to-high certainty of the estimates for several outcomes, the sample sizes were relatively small, and follow-up durations were mostly short-term. The long-term sustainability of PBMT effects is uncertain.

### Influence of PBMT parameters on treatment outcomes

Differences in PBMT protocols likely contributed substantially to the variability in outcomes observed across the included studies. Variations in wavelength, energy density, treatment duration, and application methods may have influenced the magnitude and consistency of clinical response.

Studies using near-infrared wavelengths (808–905 nm, used in five studies [28–31, 33]. Generally, studies using longer red-light wavelengths (e.g., 670 nm) demonstrated more consistent reductions in pain intensity and improvements in quality of life than studies using shorter red-light wavelengths (e.g., 670 nm) used by Ruaro et al. [34]. This may be related to the greater tissue penetration capacity of near-infrared light, allowing modulation of deeper muscular and connective tissues associated with fibromyalgia-related pain sensitization.

Variability in radiant exposure and total energy delivery may also have affected treatment response (e.g., 142.8 J/cm2 in Maciel et al.) [29]). Protocols using moderate energy delivery and multi-diode cluster systems tended to report broader improvements in pain and functional outcomes. Cluster probes delivering multiple wavelengths simultaneously [28, 30, 34]. In contrast, the Class IV high-power laser protocol demonstrated limited analgesic benefit despite substantially higher total energy delivery, suggesting that excessively high irradiance may alter the therapeutic response and involve photothermal rather than photobiomodulatory mechanisms.

Studies applying PBMT to a higher number of tender points (16–18) generally reported greater reductions in tender point count and pressure pain threshold [31, 33, 34]. Treatment duration and session frequency appeared to influence short-term outcome sustainability. Studies applying PBMT over longer intervention periods(8–12 weeks with 2–3 sessions per week) generally reported greater improvements in pain, pressure pain threshold, and functional outcomes than shorter-duration protocols [28–30]. This may indicate that repeated PBMT exposure is necessary to achieve cumulative neuromodulatory and anti-inflammatory effects in fibromyalgia syndrome.

Application techniques may have further contributed to outcome variability. Most studies used contact delivery over multiple tender points, whereas one study employed non-contact application [34]. Contact techniques appeared to produce more consistent outcomes, possibly because of improved energy transfer and reduced photon scattering. Additionally, studies treating a larger number of tender points generally demonstrated greater reductions in pain sensitivity, suggesting that wider regional coverage may be important in a condition characterized by diffuse pain distribution.

The Class IV high-power laser study [32], which used substantially higher energy delivery (> 500 mW) and total energy (4200 J/ session), produced only modest functional gains without meaningful pain relief, supporting the importance of distinguishing low-level photobiomodulation (Class III) from high-power Class IV protocols due to differing photothermal versus photomodulatory mechanisms. Furthermore, protocols combining PBMT with exercise or static magnetic fields appeared to yield superior multidimensional improvements, indicating that PBMT may be most effective as part of a multimodal rehabilitation strategy rather than as a standalone intervention.

### Comparison with previous literature

In comparison with previous literature, the current results are generally in line with the evidence supporting the analgesic and functional benefits of PBMT in chronic pain disorders. Clinical and mechanistic reviews have shown a reduction in pain and inflammation, as well as improvements in tissue metabolism and neuromodulation [16, 19, 21]. However, previous systematic reviews pointed to the large heterogeneity in PBMT dosing, wavelength, and reporting as important methodological limitations [22]. In turn, the current systematic review has also shown consistent short-term pain intensity and QoL improvements in fibromyalgia patients, but large variability in treatment parameters and outcome measures precluded meta-analysis. Hence, although the current findings are in line with the overall literature in supporting PBMT as a promising adjunct therapy, they also emphasize the need for standardized treatment protocols and reporting to improve overall comparability and the evidence base [25].

### Clinical implications

The findings of this review suggest that low-level PBMT may be considered as an adjunctive treatment option for individuals with fibromyalgia syndrome who continue to experience persistent pain, fatigue, reduced exercise tolerance, and impaired quality of life despite standard multidisciplinary care.

Current evidence suggests that PBMT may be most beneficial when incorporated into multimodal rehabilitation programs, particularly alongside exercise-based interventions. Several included studies demonstrated improvements in pain, mobility, and functional capacity when PBMT was combined with aerobic or functional exercise.

Although standardized treatment guidelines are not yet available, protocols delivering PBMT using near-infrared wavelengths (808–905 nm), applied 2–3 times weekly for 8–12 weeks across multiple tender points, appeared to produce more consistent short-term symptom improvement. Application over multiple tender points or painful muscular regions was commonly used across successful studies.

PBMT was generally well tolerated, with very limited adverse events reported. Therefore, PBMT may represent a reasonable non-pharmacological option for patients seeking adjunctive symptom management approaches, especially for those with poor tolerance or limited response to pharmacological therapies.

Nevertheless, clinicians should interpret the current evidence cautiously because of methodological heterogeneity, variability in dosimetry, and limited long-term follow-up data. PBMT should therefore currently be considered a supportive rather than a standalone intervention until larger high-quality trials establish definitive treatment recommendations.

### Strengths and limitations

Strengths of this review are PROSPERO registration, adherence to PRISMA guidelines, comprehensive databases searching, systematic assessment of bias risk, and use of GRADE assessments.

However, while the review gives some useful information on the possible use of PBMT for treating fibromyalgia, there are some important limitations that should be acknowledged. Firstly, the substantial heterogeneity in PBMT parameters (wavelength, dosimetry, treatment duration, number of sites, and session frequency) and outcome measures precluded meta-analysis and limited direct comparison across trials. This variability makes it difficult to identify optimal treatment parameters. The addition of a Class IV laser study has further increased variability in terms of mechanisms. Secondly, most included studies in this analysis had small sample sizes. This has reduced statistical power and also made it difficult to generalize the results. Moreover, Short follow-up times further limit the ability to interpret results, as fibromyalgia is a chronic condition, and the long-term sustainability and safety of PBMT remain unclear. Third, restriction of studies to English-language publications may pose a potential language bias, possibly excluding the relevant data.

Methodological issues were also observed; some trials suffered from potential bias because of insufficient blinding, missing intention-to-treat analyses, and insufficient reporting of adverse events. Because it is well known that fibromyalgia is highly sensitive to the placebo effect, blinding may be particularly important in subjective outcomes such as pain and QoL.

Finally, although proposed mechanisms include mitochondrial modulation and anti-inflammatory effects, the precise therapeutic pathways of PBMT in fibromyalgia remain incompletely understood. Randomized controlled trials with well-designed procedures and sufficient power for longer follow-up are required to enhance the evidence.

### Future directions

Future studies should focus on the following: Conducting large-scale randomized controlled trials, using standardized parameters of PBMT (wavelength, intensity, energy density, and frequency of treatment sessions), Long-term follow-up to evaluate the sustainability of treatment outcomes, comparing PBMT with guideline-based treatments, distinguishing between low-level and high-power laser modalities, and developing guidelines for dosimetry in PBMT treatment of fibromyalgia.

## Conclusion

According to this systematic review, low-intensity PBMT can be considered for providing short-term relief in pain intensity and QoL in patients suffering from fibromyalgia syndrome. Overall, PBMT was found to have good tolerability; however, there was a lack of reporting on any adverse effects within the included studies. Large variations observed in the treatment regimen and outcome measures preclude the possibility of meta-analysis. Larger and well-conducted randomized controlled trials are needed to determine optimal treatment regimens of PBMT.

## Data Availability

The datasheets & supporting documents are available from the corresponding author upon request.

## Abbreviations

FMS: Fibromyalgia Syndrome
PBMT: Photobiomodulation therapy
QoL: Quality of life
PRISMA: Preferred Reporting Items for Systematic Reviews and Meta-Analyses
LEDs: light-emitting diodes
CCO: cytochrome c oxidase
RCT: Randomized Controlled Trials
PICO: Population, intervention, comparison, outcome
GRADE: Grading of Recommendations, Assessment, Development, and Evaluation
LLLT: Low Level Laser Therapy
VAS: Visual Analog Scale
PPT: Pressure Pain Threshold
SF-36: Short Form-36
FIQ: Fibromyalgia Impact Questionnaire
FIQR: Fibromyalgia Impact Questionnaire-Revised
PBM-sMF: Photobiomodulation-static magnetic field
FSS: Fatigue severity scale
6MWT: 6 min walk test
TUG: Timed up and go test
CSPFP: Continuous Scale Physical Functional Performance
GaAlAs: Gallium, Aluminum, Arsenic
